# Activation therapy for the treatment of inpatients with depression – protocol for a randomised control trial compared to treatment as usual

**DOI:** 10.1186/s12888-019-2038-2

**Published:** 2019-02-01

**Authors:** Ian R. E. Averill, Ben Beaglehole, Katie M. Douglas, Jennifer Jordan, Marie T. Crowe, Maree Inder, Cameron J. Lacey, Christopher M. Frampton, Christopher R. Bowie, Richard J. Porter

**Affiliations:** 1 Department of Psychological Medicine, University of Otago, PO Box 4345, Christchurch, 8140 New Zealand; 2 Department of Psychology, Queen’s University, Kingston, Canada; 3 Specialist Mental Health Services, Canterbury District Health Board, Christchurch, New Zealand

**Keywords:** Depression, Therapy, Cognitive activation, Activation therapy, Inpatient, Actigraphy, Neurocognitive, Rehospitalisation rate

## Abstract

**Background:**

Inpatients with depression have a poor long term outcome with high rates of suicide, high levels of morbidity and frequent re-admission. Current treatment often relies on pharmacological intervention and focuses on observation to maintain safety. There is significant neurocognitive deficit which is linked to poor functional outcomes. As a consequence, there is a need for novel psychotherapeutic interventions that seek to address these concerns.

**Methods:**

We combined cognitive activation and behavioural activation to create activation therapy (AT) for the treatment of inpatient depression and conducted a small open label study which demonstrated acceptability and feasibility. We propose a randomised controlled trial which will compare treatment as usual (TAU) with TAU plus activation therapy for adult inpatients with a major depressive episode. The behavioural activation component involves therapist guided re-engagement with previously or potentially rewarding activities. The cognitive activation aspect utilises computer based exercises which have been shown to improve cognitive function.

**Discussion:**

The proposed randomised controlled trial will examine whether or not the addition of this therapy to TAU will result in a reduced re-hospitalisation rate at 12 weeks post discharge. Subjective change in activation and objectively measured change in activity levels will be rated, and the extent of change to neurocognition will be assessed.

**Trial registration:**

Unique trial number: U1111–1190-9517.

Australian New Zealand Clinical Trials Registry (ANZCTR) number: ACTRN12617000024347p.

## Background

Depression is the most prevalent mental disorder and the leading cause of disability worldwide [1]. In its most severe form, depression causes severe morbidity and may require inpatient treatment. Patients requiring admission, compared with those able to be treated as outpatients, are more likely to be suicidal, have psychomotor retardation, reduced interest in work and activities [2], and have significant neurocognitive dysfunction [3]. Patients who are hospitalised for depression also have a very poor longer-term outcome, with a high rate of completed suicide (7% in 15 years with the highest rates soon after discharge [4, 5]), high ongoing morbidity (12% incapacitated over 15 years [4]) and relapse, with only 20% of patients remaining continuously well over 15 years [4, 6].

In Christchurch, New Zealand, between 2011 and 2016, approximately 300 patients per year were admitted to hospital with a primary diagnosis of major depression. The typical length of admission was 2–3 weeks. Following discharge, 40% of patients were re-admitted to the inpatient unit within 12 weeks and a further 7% between 3 and 6 months. Re-admission rates are of concern internationally with governments in several countries now setting benchmarks for reducing early re-admission, which is seen as an indicator of the quality of inpatient care [7–9]. During the three months after discharge from psychiatric inpatient units, the risk of completed suicide is 100 times the global base rate [10], further suggesting the need for enhanced inpatient care. The inpatient period is therefore vital and provides an opportunity for intensive pharmacological and psychological treatment to be provided [11–13].

### Limitations of existing inpatient treatment of depression

Current clinical practice involves short stabilisation admissions with most psychotherapeutic treatment (if available) occurring on an outpatient basis following admission. In this context, acute inpatient services have often been criticised for failing to provide adequate therapeutic environments [14] and psychological/behavioural treatment [15]. Surveys of mental health inpatients consistently highlight concerns about inpatient environments including a lack of people to talk to, an over-reliance on medication, and boredom [16–19]. In many cases, the sole focus of psychiatric inpatient treatment has been safety and crisis stabilization rather than psychotherapeutic benefit [20]. If acute inpatient services are to meet government benchmarks, there is an urgent need for effective interventions that can balance the need for containment and risk management with the therapeutic needs of patients [21]. This provides a powerful argument for research and interventions that seek to improve treatment of inpatient depression. Our review of the evidence for the effectiveness of non-pharmacological interventions for acutely depressed inpatients [22] and our feasibility study of an intensive psychological therapy reported here show that it is feasible to deliver psychological/behavioural interventions in a busy inpatient setting and that these could easily be undertaken by inpatient nurses with a manageable amount of training. Training in an evidence based intervention is also likely to have a beneficial effect on staff morale and expertise [23, 24].

### Neurocognitive dysfunction in mood disorders

Neurocognitive dysfunction (e.g. in the areas of memory, planning, organisation, psychomotor speed) in mood disorders is an important and disabling feature [25, 26]. It is increasingly recognised that in recurrent unipolar major depressive disorder (MDD) and in bipolar disorder, neurocognitive dysfunction persists despite relatively complete resolution of mood symptoms [26, 27]. Studies also suggest a limited benefit of traditional pharmacological treatment on neurocognitive dysfunction in MDD [3, 28, 29].

Neurocognitive deficits are larger and also fail to improve significantly with treatment in inpatients with depression [3, 30]. Our study of inpatients in Christchurch suffering from a MDE showed effect size differences from healthy control participants of 0.8–1.0 in most aspects of neurocognitive functioning [3]. After 6 weeks of standard treatment, half of this sample had 50% or greater improvement on mood rating scales, and 80% had been discharged, yet there was no evidence of improvement in neurocognitive function. On an individual level, global neurocognitive performance was more than 0.5 SD below controls in 77% of patients and more than 1 SD below controls in 49% of patients [3]. Impairment in neurocognitive function is linked to poor global psychosocial adjustment, occupational difficulties, and interpersonal problems [29, 31]. In inpatients with depression, executive function correlated highly with social and occupational functioning [32], while Jaeger et al. [33] reported a correlation between neurocognitive measures and disability 6 months after hospitalisation for MDD. Preliminary data also suggest that neurocognitive impairment increases relapse rates [34, 35].

### Evidence for a benefit of cognitive activation treatments in depression

Neurocognitive deficits are recognised as an important target for treatment in mood disorders [36–39] with traditional pharmacological treatments being relatively ineffective in reversing neurocognitive impairment [29, 40]. Traditional psychological treatments also show little evidence of a positive effect on cognitive function in depression [41]. However, metacognitive therapy (MCT), a newer therapy focusing on metacognitive processes and strategies including cognitive control and attention, incorporates regular practice of an Attentional Training Technique. In a randomised controlled outpatient trial comparing MCT with cognitive behavioural therapy (CBT) for depression, we showed no significant difference in reduction of depression symptoms [42] but a significant advantage of MCT over CBT on measures of executive function, with effect sizes of *d* = 0.5–0.7 [43]. This suggested that incorporation of cognitive focus and practice may begin to address the problem of neurocognitive deficit.

Studies involving training in, and practice of, cognitive exercises have varied from using repeated practise of limited tasks (usually termed cognitive training or activation) to more rehabilitative approaches using a wider range of tasks and additional strategy coaching (often termed cognitive remediation) [44]. Studies have been small and preliminary [45–47] but meta-analysis has suggested moderate effects on daily functioning [48].

One justification for cognitive activation (CA) is to attempt to reverse neurobiological abnormalities in depression. Neuroimaging studies in depression suggest there is decreased recruitment of executive control networks, including regions such as the dorsolateral prefrontal cortex (DLPFC) [49]. Siegle et al. [50] examined the utility of repeated practise on two tasks hypothesised to activate DLPFC, in outpatients with severe depression. Fifteen patients received six cognitive training sessions, utilising these tasks, over a 2 week period, while seven received care as usual. The cognitive training group showed significantly decreased self-rated depression score and reduced rumination. Six patients completed functional magnetic resonance imaging (fMRI) before and after cognitive training, with preliminary evidence of increased activity in the DLPFC. The study therefore presents preliminary evidence of clinical efficacy and concomitant expected biological changes. In a later replication, patients undergoing intensive day hospital treatment for depression, who received similar intensive cognitive training, required only 30% as many days of intensive day hospital treatment in the subsequent year as a service control group who did not receive this intervention [51].

Evidence of a benefit of cognitive training in inpatients with depression was provided by a study which randomly assigned inpatients to 4 weeks treatment as usual or 12 sessions of cognitive training (involving a more rehabilitative approach). The latter group showed significantly greater improvement in verbal and non-verbal memory, executive function and working memory (ES 0.52–0.98) [52].

### De-activation in depression

Psychomotor slowing has long been seen as a core feature of severe or melancholic depression [53] and is therefore particularly prevalent in inpatients [2]. Uher et al. [54] examined the course and impact of the cluster of de-activation symptoms (loss of interest, diminished activity, indecisiveness and lack of enjoyment) showing that these residual symptoms strongly predicted poor treatment outcome in two large samples of depressed patients. The predictive effect of the de-activation symptoms was found to be independent of overall depression severity and antidepressant treatment, and authors suggested that if present, these symptoms may merit specific treatment in addition to antidepressants. Reduced energy has also been identified as a common, persisting residual symptom in patients remitted from a MDE [55–57] and de-activation has been shown to predict poor response to treatment in bipolar depression [58].

### Behavioural activation in depression

Behavioural Activation (BA) addresses inactivity and avoidance (symptoms of de-activation) in depression by assisting patients to engage in previously or potentially rewarding and valued life activities using scheduling of pleasant and mastery events [59]. In outpatients with depression, studies show large effect size differences compared with no treatment and no significant difference from CBT or other evidence based comparators [60–63]. Compared with antidepressant medication, meta-analyses have reported BA to be superior in improving acute symptoms of depression, regardless of initial severity of depression [64].

In severe depression, BA has pragmatic advantages over other more complex and demanding psychotherapies, because it can be delivered in the short timeframe of a typical inpatient admission. BA has, in fact, shown superiority over CBT in severely depressed patients [65].

Our review of non-pharmacological treatments for inpatients with depression suggests promise for the use of BA in inpatient settings [12]. In addition ultra-brief BA has recently been trialled in inpatient settings with positive results in several small studies [66, 67].

### Development of activation therapy

To address symptoms of de-activation (including loss of interest and enjoyment, reduced activity, and indecisiveness) and the significant neurocognitive impairment of this group of patients, we developed Activation Therapy (AT). This combines CA and BA, to complement existing treatments for inpatient depression and provide targeted psychological treatment for both activation and cognitive deficits in those with severe depression.

The rationale for using a combined therapy is as follows:

1. Patients in this study will be severely depressed. We do not believe that they will engage in CA without the support of a broader psychological treatment (BA), and BA will be facilitated by improving the cognitive capacity to engage in activities (CA).
2. Evidence from schizophrenia suggests better effects of CA when combined with other psychotherapy, rather than alone [68].
3. A partial cause of neurocognitive impairment is assumed to be avoidance of cognitive activities which are perceived as “difficult” and so avoidance becomes negatively reinforcing (i.e. the person gets relief from frustration or a sense of failure by avoiding the activity). Attempting to reverse this process will require therapeutic input beyond a simple focus on “practice” of these activities and will be targeted by BA. BA enhances resilience by including the coaching of approach strategies (e.g. trial and error, problem solving) as alternatives to withdrawal or avoidance in the face of frustration or potential failure.

### Cognitive activation component

During AT, patients and therapists use a computerised cognitive practise programme (Scientific Brain Training Pro (SBT-pro)) [69]. SBT-pro provides user friendly computer games or tasks designed to remediate typical cognitive deficits associated with depression. There is flexibility to alter the level of difficulty to meet the individual’s current functioning and incrementally increase task difficulty as cognition improves. Therapists select cognitive tasks suitable for the patient’s current level of functioning at study commencement, and in subsequent sessions, review progress. Collaborative practice, encouragement and coaching takes place.

In contrast to cognitive remediation, the focus of cognitive activation is on stimulation and training in a more independent training environment, thus lessening the burden on staff resources.

The computerized training includes an algorithm to adapt the difficulty level of training to maintain success rate at 80 to 90% to optimise motivation in severely depressed patients. This provides sufficient levels of success with the still tolerable levels of challenge needed to promote neuroplasticity and to activate important brain regions.

### Behavioural activation component

This component is drawn from Lejuez et al. [59]. BA involves identification of positive and negative reinforcers maintaining depressive symptoms and behaviours, and of reinforcers that promote more adaptive behaviours. Personal values are identified and activities focussed on these values are selected. Therapy sessions include goal-setting, activity monitoring and scheduling of pleasant and mastery events between sessions. Close monitoring of progress and collaborative problem-solving of potential difficulties occurs.

### Practice component

Between therapist sessions, patients are encouraged to log onto their own CA online account to practise cognitive games. Therapists monitor this activity on a daily basis online and add comments between sessions when necessary. BA involves daily practise of scheduled activities, including scheduled online CA practice activities.

### Feasibility of AT and pilot study outcomes

We conducted an open label pilot study of AT to assess 1) the feasibility of recruiting and delivering AT in an inpatient setting, 2) the acceptability to participants, and 3) preliminary data to inform power calculations for a larger trial. The trial was registered and had ethical approval (University of Otago Human Ethics Committee (Health) H15/056. The aim was to deliver up to nine sessions of AT (in addition to TAU), with daily CA practice recommended over a three week period.

Twenty four participants were recruited and 19 completed both baseline and follow up measures. It was possible to engage patients and begin therapy within 3 days of admission.

Patients completed a mean of 6.2 of a planned 9 therapy sessions and 5.3 online CA practice sessions (mean total 115 min) in 3 weeks. All patients said they would recommend AT to others with depression and 79% were “satisfied” or “very satisfied” with treatment. Change in activity levels monitored by actigraphy indicated that the change (gradient) of activity correlated closely with change in clinical rating scale measures and change in functioning [70]. All patients improved significantly with pre-post clinical measures indicating significant improvement for depression (Quick Inventory of Depressive Symptomatology (QIDS) [71] (mean change − 9.3 points) and Clinical Global Impression: Severity (CGI -S) [72] (mean change − 1.5 points) and functioning (Functioning Assessment Short Test (FAST) [73] (mean change − 11.6 points). These pilot results combined demonstrated that it is feasible to deliver AT in an inpatient setting, inpatients found it acceptable and the promising preliminary efficacy data all justify this proposed randomized controlled trial.

The feasibility aspect identified several possible improvements to delivery of AT in this setting. Firstly, the initial engagement/values /goal-setting module appeared too complex and time consuming for severely depressed patients. This module has therefore been simplified. Secondly, many people were discharged during the 3 weeks after which it was not easy to complete the programme. It was identified that the programme could have been more intensive with more sessions per week. The proposed AT will therefore be shorter and more intensive - 8 sessions of 30–40 min individual therapy and 10 sessions of 20–40 min online practice within 2 weeks.

## Methods/Design of Proposed trial

### Randomised controlled study of two weeks activation therapy versus treatment as usual for the treatment of inpatient depression

This randomised controlled trial evaluates the effectiveness of AT by comparing TAU with TAU plus AT for depressed inpatients.

A Standard Protocol Items; recommendation for Interventional Trials (SPIRIT) [74] schedule of enrolments, assessments and interventions is presented in Table 1 and a SPIRIT checklist is provided in Additional file 1.

**Table 1:** Schedule of enrolments, assessments and interventions

	Enrolment	STUDY PERIOD						
		Allocation		Post-allocation				
TIMEPOINT**	*−3 to 0 days*	0	*0*	*1 week*	*2 weeks*	*8 weeks*	*14 weeks*	*1 year*
ENROLMENT:								
1) Eligibility screen	X							
2) Informed consent	X							
3) Baseline measures (TAU)		X						
4) Baseline measures (AT)		X						
5) Allocation	X							
INTERVENTIONS:								
Activation therapy			X	→	X			
Treatment as usual			X	→	X			
ASSESSMENTS:								
Actigraphy			X	→	X			
Cognitive assessments			X				X	
FAST & QoL.BD			X				X	
MADRS & BDI-2			X		X	X	X	
Days spent in hospital								X
Readmissions							X	X

*Abbreviations*: *TAU* treatment as usual, *AT* activation therapy, *FAST* functioning assessment short test, *QoL.BD* quality of life in bipolar disorder questionnaire, *MADRS* Montgomery-Asberg Depression Rating Scale, *BDI-2* Beck Depression Inventory - Second Edition

The primary hypothesis is that rates of re-admission to hospital in the 12 weeks following discharge will be significantly less in the AT group compared with those receiving TAU. (We note that discharge is after a median of 2 weeks, but is variable – so that the 12 weeks following discharge referred to in the primary outcome will not always align with the 14 weeks from baseline of the most secondary outcomes).

### Secondary hypotheses

- AT will result in significantly greater increase in objective activity levels as measured by actigraphy (baseline to 2 weeks) compared with TAU and greater increase in self-reported activation levels (baseline – 2 and 14 weeks).
- AT will result in significant improvement in objective global neurocognitive function, and in self-reported cognitive function, compared with TAU (baseline - 14 weeks) as assessed by the Cognitive Complaints in Bipolar Disorder Rating Assessment (COBRA) [75].
- General functioning measured on the FAST [73] will show significantly greater improvements at follow-up in patients receiving AT compared with those receiving TAU (baseline - 14 weeks).
- Patients receiving AT will have significantly greater improvement on the Montgomery-Asberg Depression Rating Scale (MADRS) [76] and on the Beck Depression Inventory - Second Edition (BDI-2) [77] scale at follow-up (baseline - 14 weeks).
- Rates of re-admission to hospital in the 12 months following discharge will be significantly less in the AT group compared with those receiving TAU.
- Total days of hospitalisation during the 1 year from baseline will be fewer in the AT group and costs will be less when cost of AT and days in hospital are taken into account.

### Sample

We will recruit 170 inpatients aged 18–65 years admitted with a primary DSM-5 [78] diagnosis of major depressive episode (unipolar or bipolar), who are computer literate, able to complete questionnaires and therapy in English, and willing to provide informed consent. Based on our pilot study and hospital admission rates we expect to recruit the cohort from 3 local hospitals, 100 from Te Awakura - Adult Acute Inpatient Service, Hillmorton Hospital, Christchurch, and 35 each from Manaakitanga Mental Health Unit, Grey District Hospital, Greymouth, and Kensington Centre, Timaru. Te Awakura is the sole provider of acute adult psychiatric inpatient services to slightly over 500,000 people in and around the city of Christchurch. It provides 64 inpatient beds over 4 wards and like the other two is part of a comprehensive, free, government run, mental health service. The Manaakitanga Mental Health Unit provides for a geographically large area with a relatively small rural and semi-rural population (total population approximately 32,000) of the west coast of the South Island. The Kensington Centre provides acute inpatient services to the largely agricultural area surrounding Timaru on the east coast.

Exclusion criteria will be minimal in order for the trial to be generalisable to inpatient populations with depression and will be: primary diagnosis of schizophrenia or current severe drug or alcohol misuse, co-morbid serious endocrinological, neurological or chronic medical conditions, pregnancy, previous serious head injury, having had electroconvulsive therapy (ECT) in the past 6 months or planning ECT for the current admission. All patients will be treated with pharmacotherapy as deemed appropriate by the treating multi-disciplinary team and randomised to receive additional AT or no additional therapy (TAU).

### Recruitment process

Patients will be screened by treating clinicians (nurses or psychiatrists) within 1–2 days of admission. Brochures describing the study will be available to potential participants. If the patient agrees, a member of the research team will meet potential participants to assess eligibility, discuss the study, obtain written consent and perform a short version of the Structured Clinical Interview for DSM-5 (SCID5) [79] to confirm depression and ensure exclusions have been ruled out. The reason given by those who do not want to participate in the study along with some basic anonymised demographic data will be recorded to provide an indication of any cohort sample bias (according to CONSORT guidelines) [80]. Following assessment and consent, a research assistant (RA) will undertake baseline measures after which participants will be randomised. The intervention will then be commenced as soon as possible. We aim to complete the process and begin therapy within 3 days of admission.

### Randomisation

This will be achieved by computer generated permuted block, stratified by centre and by mood disorder diagnosis (bipolar vs unipolar). Randomisation will occur through the RA accessing a password protected on-line system.

### Blinding

It is not feasible to blind patients or therapists. However the outcome measures will be administered by an RA who will be blind to treatment. At the start of each assessment the RA will explain the importance of not revealing which arm they have been in. The RA will administer the main outcome measures – cognitive testing, functional assessment and mood rating scales.

### Intervention

All patients will be treated with pharmacotherapy and other usual ward interventions as deemed appropriate by the treating multi-disciplinary team whether receiving active treatment (AT) or TAU. Active AT treatment will consist of 8 × 30–40 min therapist sessions of AT and a recommended 10 x online practice sessions over 2 weeks. This will be in addition to TAU described below. Therapists will be experienced registered nurses, social workers, or clinical psychologists familiar with the requirements of ward environments and working within multi-disciplinary teams. Group supervision will occur over the course of the study.

Overall clinical responsibility remains with the treating team and cessation of therapy can occur on their request. Although it is not anticipated that the intervention will cause significant harm, any instances of this occurring will be recorded along with any reasons for cessation of therapy. Decisions regarding discharge remain the responsibility of the treating team although if patients are discharged prior to receiving two weeks of therapy, therapy can be continued in the outpatient setting to avoid any reduction in dose.

### Training/adherence

AT will be delivered according to our AT manual, by mental health professionals trained and closely supervised with a focus on adherence and competence. Therapists will complete fidelity checklists of core strategies after each session to ensure all elements are delivered. A random selection of 10% of audiotaped sessions will be listened to by a supervisor and scored according to the Quality of Behavioral Activation Scale [81]. Participant adherence will be measured by the 9-item Behavioral Activation for Depression Scale - Short Form [82] completed by participants at baseline, session 4 and session 8. A single question self-rating the extent of practice of BA activities within the past 24 h will be completed by participants at each session. The frequency and duration of online CA exercises completed between therapy sessions will also be monitored through the use of the SBT-pro programme.

### Control treatment

The control treatment will be TAU. This involves nursing care, regular review with medical staff, pharmacotherapy, and an occupational therapist led therapeutic programme consisting of educational, diversional, and therapeutic group activities. Availability of psychology input is very limited due to resourcing factors and usually only occurs in cases in which the admission has become prolonged and would therefore occur after the end of the treatment period.

### Primary outcome measure

Rates of hospital re-admission within 12 weeks of discharge will be determined from the patient’s electronic hospital record and it is therefore very unlikely that there will be any missing data for this outcome.

#### Power

The 12 week re-admission rate between 2013 and 2015 (prior to the start of the feasibility study) was 40%. We propose that a clinically significant reduction in this rate would be to 20%. To have 80% power to show such a difference between groups as statistically significant (*p* < 0.05) will require 82 patients per group.

The choice of a reduction to 20% is based on the following. The three month re-admission rate of patients in the open label feasibility study was 17% compared with 40% prior to the study. In addition, although this reduction represents a halving of the re-admission rate, the number needed to treat (NNT) to prevent one re-admission would be 5 and we do not believe that a higher NNT would provide sufficiently convincing evidence to proceed with roll out.

The fact that 60% of patients would not have been re-admitted is important. They represent a potential dilution of the primary outcome. However, we hypothesise that there will be benefits in these patients in the secondary outcomes, in particular in activation and neurocognitive function.

#### Analysis

The primary outcome will be analysed by Cochrane Mantel-Haenszel Test, stratified by site and mood disorder diagnosis. The analysis population will be intention-to-treat.

#### Sensitivity analysis (per protocol)

This will include only participants who complete a pre-defined proportion of treatment. Per protocol = at least 5/8 sessions with therapist plus at least 6/10 online practise sessions of at least 15 min each.

### Secondary outcomes

While the study is primarily a pragmatic trial with a clinical outcome, it is important to examine the mechanism of change - to measure both change in de-activation and neurocognitive function, both objectively and subjectively, and to measure general functioning and quality of life.

#### Change in overall activity levels

Activity levels will be recorded continuously as activity counts using a triaxial actigraph (‘Motionwatch 8’ (MW8) by CamNtech Ltd., UK). Participants from both TAU and TAU plus AT groups will be asked to wear this day and night for the AT administration period of the study (2 weeks). The MW8 is waterproof, durable, and will not need to be recharged during this period. Data is not visible to the wearer (avoiding possible motivating effect) and is uploaded from the MW8 after removal at 2 weeks. Average activity counts per epoch per day will be used to calculate change in activity levels over the first and second weeks.

#### Subjective change in “activation”

Items from the MADRS, in addition to the Beck Depression Inventory, 2nd version (BDI-II - see below) will be examined. These items are determined from a previous factor analysis and analysis of residual and predictive items, as per Uher et al. [54].

#### Change in neurocognitive function

##### Neurocognitive test battery

The main outcome will be change in a global neurocognition score generated from a preselected group of neurocognitive measures covering the domains of visual and verbal memory and learning, attention, executive function, and psychomotor function. The choice of measures is based on those used in previous related studies [3, 83, 84]. To minimize practice effects the cognitive test battery is selected to avoid tasks directly analogous to the practice exercises in the CA battery. *The composite score is comprised of the following variables:*

- Groton Maze Learning Test (Cogstate) [85] – total errors in all learning trials
- Groton Maze Learning Test (Cogstate) [85] – total errors in delayed trial
- Rey Auditory-Verbal Learning Task [86] - total words recalled in all learning trials
- Rey Auditory-Verbal Learning Task [86] – total words recalled on delayed recall trial
- Category Fluency [87]- total words generated
- Category Fluency – Switching [87] - total correct switches
- Digit Span [88] - total forwards and backwards
- Timed Chase Test (Cogstate) [85] – number of correct moves per second
- Digit Symbol Substitution Test [88] - total correct symbols generated

Neurocognitive variables will be grouped to fit into one of four pre-defined neurocognitive domains (1) verbal learning and memory, 2) visuospatial learning and memory, 3) executive function/attention, 4) psychomotor speed). Domain scores will be calculated by averaging Z-scores of tests (test score/SD of the score at baseline) within each domain. The composite score will be calculated by averaging Z-scores across the four domain scores [39, 89].

#### Subjective assessment of neurocognitive impairment

Cognitive Complaints in Bipolar Disorder Rating Assessment (COBRA) has been developed specifically for use in mood disorders [75, 90, 91].

#### General functioning

The FAST will be used to assess functioning at baseline and 14 weeks. The FAST has a close correlation with scores on the Global Assessment of Functioning Scale, which has been very widely used and validated in psychiatry, however, the FAST is more detailed and includes specific assessment of cognition. We used this scale in our pilot study and developed training with reference to the original developers of the scale, using their manual [73].

#### Mood rating scales

The Montgomery Asberg Depression Rating Scale (MADRS) is a clinician-rated depression severity scale which will be administered at baseline, 1 and 2 weeks post baseline, and again at 6 and 12 weeks post discharge.

The Beck Depression Inventory, Second Version (BDI-2) is a subjectively rated depression scale to be administered at the same time points.

#### Re-admission/days in hospital/economic analysis

Patients will be followed for 1 year post-treatment to examine a) re-admission to hospital with a primary diagnosis of depression and b) total days in hospital, including during the index admission, and c) time in Specialist Mental Health Services outpatient care. Total days in hospital will not be normally distributed with a small number of patients experiencing protracted index or repeat admissions.

### Secondary analyses

#### Analysis of secondary outcomes

Analysis will use a general linear mixed model with treatment and stratification factors (site and mood disorder diagnosis) as between-participant factors and time (baseline/follow-up) as a within-participant factor. Analysis population will be intention-to-treat with all patients who are randomised entered into the analysis. Participants who do not undergo repeat assessment will be assumed not to have changed. Close liaison with patients and their clinicians following discharge and an interim contact to carry out mood ratings, plus testing in patients’ homes, will maximise follow-up and minimise the chance of differential dropout between groups.

The analysis of individual domains of cognitive function will guide future development of therapy.

#### Completers analysis

The completers analysis will be as per primary analysis but including only the population who complete both cognitive testing sessions irrespective of treatment adherence.

#### Per protocol analysis

The per protocol analysis will include only participants who complete a pre-defined proportion of treatment. Per protocol = 5/8 sessions with therapist plus 6/10 online practise sessions of at least 15 min each.

#### Comparison of re-admission rates

This will be undertaken with a Chi squared test given the expected distribution of readmission rates.

#### Exploratory analysis

The exploratory analysis is to examine the effects of mood disorder diagnosis (bipolar vs unipolar), dose of therapy, dose of practice, baseline severity of depression, baseline severity of functional impairment on the relative effects of the two treatments.

### Data management

An independent Data Safety and Monitoring Committee has been set up and will conduct 6-monthly meetings facilitated by and with data prepared by a member of the research team (Professor Chris Frampton) The committee will consist of an international expert in psychotherapy trials, a New Zealand expert in Psychiatry clinical trials, and a NZ biostatistician.

### Protocol modification

Any substantive modification to the study protocol, particularly those related to patient selection, treatment, data collection, and study outcomes will be discussed by the study group and submitted to the Health and Disability Ethics Committees (New Zealand) for consideration. Any approved modification will be recorded on the ANZCTR and noted in any papers submitted for publication.

## Discussion

The pilot study indicated acceptability and feasibility of the treatment and sufficient recruitment to support an adequately powered randomised controlled trial (RCT). As noted it also suggested some modifications to the original protocol and to details of the therapy which have informed the design of the proposed RCT. The pilot study showed that patients receiving AT improved on depression rating scales and in functioning, however, the same improvement may have occurred with standard care and without the addition of AT so no conclusions regarding the effectiveness of AT can be drawn.

In designing the protocol for the RCT, various issues have required consideration as follows.

### Choice of comparator treatment

It could be argued that any input involving 8 sessions with supportive therapists is likely to be effective and that a more active control condition than TAU would therefore be appropriate. However, TAU represents standard care for most inpatient units and the study was conceived to trial a treatment designed to address the clinically significant residual cognitive impairment which we observed in our previous study [3]. We therefore believe that it is appropriate to compare our “best possible package” with current treatment.

### Choice of primary outcome measure

There are five outcome measures of particular importance in this study – hospital re-admission rates, change in activity levels, change in cognitive function, change in general functioning (social, occupational, interpersonal), and change in mood symptoms. The choice of re-admission rates as primary outcome is based on the following.

1. High re-admissions rates are internationally recognised as an indicator of the quality of inpatient care and reduction constitutes a Key Performance Indicator in NZ [9] and in other countries.
2. Re-admission soon after discharge has a significant impact on patients, disrupting social and occupational re-integration.
3. In depression, re-admission usually indicates an increase in risk of suicide, an outcome which is vastly increased in risk in the 3 months following admission [10]. Re-admission rates are therefore indicative of level of risk in this group.
4. Re-admission rate is easily understood by and communicated to clinicians and managers and provides a good basis to proceed to evaluations of introduction of AT into clinical practice if the study shows an advantage on this outcome.

### Duration of treatment

In the context of the usual course of severe depression, this intervention is short. However, because of hospitalisation, patients are not only cut off from usual sources of activity and support but also have time during which to undertake this intensive therapy. The therapy also targets the worst phase of the illness and may shorten this and improve functioning at a particularly vulnerable time. The point of discharge is associated with a sudden return to normal roles (interpersonal, social and occupational), all of which may be particularly stressful in the context of severe ongoing cognitive impairment. This is a high risk period for relapse and suicide [10]. It is therefore important to target this problem rapidly and intensively.

### Possible uptake of therapeutic techniques into control arm

Staff in the unit will be aware of the techniques being used in AT and if they adopted these, this could dilute the effect of the intervention. However, staff do not have protected time, are not fully trained in BA and neither they nor the patients in the TAU arm will have access to the CA computer package. We therefore believe that this dilution will be minimal.

In conclusion, we have demonstrated the feasibility of providing AT in an inpatient setting. We have also outlined our rationale and proposal for a RCT assessing AT compared with TAU for the treatment of cognitive impairment in severe inpatient depression.

## Abbreviations

AT: Activation therapy
BA: Behavioural activation
BDI -2: Beck Depression Inventory - Second Edition
CA: Cognitive activation
CBT: Cognitive behavioural therapy
CGI-S: Clinical Global Impression: Severity
COBRA: Cognitive Complaints in Bipolar Disorder Rating Assessment
DSM-5: Diagnostic and Statistical Manual of Mental Disorders, fifth edition
ECT: Electroconvulsive therapy
ES: Effect size
FAST: Functioning assessment short test
fMRI: Functional magnetic resonance imaging
MADRS: Montgomery-Asberg Depression Rating Scale
MCT: Metacognitive therapy
MDD: Major depressive disorder
QIDS: Quick Inventory of Depressive Symptomatology
QoL.BD: Quality of Life in Bipolar Disorder questionnaire
RA: Research assistant
RCT: Randomised Controlled Trial
SBT-pro: Scientific Brain Training Pro
SCID5: Structured Clinical Interview for DSM-5
SD: Standard deviation
SPIRIT: Standard Protocol Items; recommendation for Interventional Trials
TAU: Treatment as usual
